# Transcriptomic analysis of nitrogen metabolism pathways in *Klebsiella aerogenes* under nitrogen-rich conditions

**DOI:** 10.3389/fmicb.2024.1323160

**Published:** 2024-02-28

**Authors:** Yanyan Chen, Yijing Lin, Jingyi Zhu, Jiayin Zhou, Haoyi Lin, Yiting Fu, Yan Zhou

**Affiliations:** Life Science and Technology School, Lingnan Normal University, Zhanjiang, China

**Keywords:** *Klebsiella aerogenes*, nitrogen, nitrate assimilation, metabolism, differentially expressed genes

## Abstract

The acceleration of the nitrogen cycle and the nitrogen excess observed in some coastal waters has increased interest into understanding the biochemical and molecular basis of nitrogen metabolism in various microorganisms. To investigate nitrogen metabolism of a novel heterotrophic nitrification and aerobic denitrification bacterium *Klebsiella aerogenes* strain (B23) under nitrogen-rich conditions, we conducted physiological and transcriptomic high-throughput sequencing analyses on strain B23 cultured on potassium nitrate–free or potassium nitrate–rich media. Overall, *K. aerogenes* B23 assimilated 82.47% of the nitrate present into cellular nitrogen. Further, 1,195 differentially expressed genes were observed between *K. aerogenes* B23 cultured on potassium nitrate–free media and those cultured on potassium nitrate-rich media. Gene annotation and metabolic pathway analysis of the transcriptome were performed using a series of bioinformatics tools, including Gene Ontology, Kyoto Encyclopedia of Genes and Genomes, and Non-Redundant Protein Database annotation. Accordingly, the nitrogen metabolism pathway of *K. aerogenes* B23 was analyzed; overall, 39 genes were determined to be involved in this pathway. Differential expression analysis of the genes involved in the nitrogen metabolism pathway demonstrated that, compared to the control, *FNR*, *NarK*/*14945*, *fdx*, *gshA*, *proB*, *proA*, *gapA*, *argH*, *artQ*, *artJ*, *artM*, *ArgR*, *GAT1*, *prmB*, *pyrG*, *glnS*, and *Ca1* were significantly upregulated in the nitrogen-treated *K. aerogenes* B23; these genes have been established to be involved in the regulation of nitrate, arginine, glutamate, and ammonia assimilation. Further, *norV*, *norR*, and *narI* were also upregulated in nitrogen-treated *K. aerogenes* B23; these genes are involved in the regulation of NO metabolism. These differential expression results are important for understanding the regulation process of key nitrogen metabolism enzyme genes in *K. aerogenes* B23. Therefore, this study establishes a solid foundation for further research into the expression regulation patterns of nitrogen metabolism–associated genes in *K. aerogenes* B23 under nitrogen-rich conditions; moreover, this research provides essential insight into how *K. aerogenes* B23 utilizes nutritional elements.

## 1 Introduction

Marine microorganisms are diverse, abundant, and widely distributed throughout marine ecosystems. These microorganisms act as both producers and consumers, making significant contributions to primary productivity and biomass within the ocean (Fuhrman and Hagström, 2008). However, the marine environment presents a complex and varied set of environmental characteristics across different regions, which have been attributed to both natural factors and human activities; these characteristics include high salinity (Xu et al., 2023), high and low temperatures (Cheung et al., 2021; Kang et al., 2021), and oligotrophic conditions (Duarte et al., 2013). Microorganisms can adapt and survive within complex marine environments; ultimately, this can lead to the formation of unique community structures and distribution characteristics. Microorganisms contribute to almost all biochemical reactions and biogeochemical cycles within the ocean. Therefore, understanding their community composition, structure, and distribution across different habitats, as well as elucidating their specific metabolic characteristics, becomes necessary for developing an in-depth understanding of the ecological functions of marine microorganisms.

Nitrogen is an essential element for life and a crucial component of biogeochemical cycles in aquatic ecosystems (Stüeken et al., 2016). However, the acceleration of the nitrogen cycle, owing to increased industrial activity and the extensive use of nitrogen fertilizers, has led to significant and challenging environmental issues that have a direct impact on human populations worldwide. Microbial nitrogen assimilation primarily occurs via the conversion of nitrate into ammonia by a series of enzymes such as nitrate reductase and nitrite reductase (Matassa et al., 2016). These microorganisms then use this ammonia for amino acid synthesis and transformation (Mena-Rivera et al., 2023). Amino acids are primarily used to synthesize proteins that can then be modified, sorted, transported, and stored in microbial organisms (Xu et al., 2015). This process is coordinated with microbial carbon metabolism and is a fundamental pathway for microbial life. Prior research has indicated that most oceans are currently experiencing nitrogen limitation (Buchanan et al., 2021). In recent years, with increasing marine biogeochemical research, the biochemical and molecular basis of nutrient metabolism in various microorganisms has become a key subject of interest (Kuypers et al., 2018). Studying nitrogen metabolism can, ultimately, improve our understanding regarding the utilization of nitrogen nutrients by marine microorganisms; in turn, this can contribute to the protection of the marine environment and promote the healthy development of the aquaculture industry. Studies on microbial-mediated marine nitrogen cycling have employed techniques such as isotope tracing (Glaze et al., 2022), quantitative PCR (Mosley et al., 2022), and metagenomics (Sun and Ward, 2021); these approaches have been instrumental in investigating how microorganisms absorb and utilize different nitrogen sources in different marine habitats at the physiological, genetic, and protein levels. In recent years, Archaea (Kirchman, 2012), γ-proteobacteria (Ravcheev et al., 2005) and *Bacillus* (Yang et al., 2021) have been isolated from seawater and have, ultimately, been determined to participate in various biological processes such as the metabolism of urea, reduction of nitrate and nitrite, and denitrification. Fungi, Sphingomonadales, and Pseudomonadales play important roles in ammonia oxidation, nitrate assimilation, and nitrification processes in the deep sea (Hawley et al., 2014; Li et al., 2018).

Overall, this study focused on *Klebsiella aerogenes* B23, which was newly isolated from a marine aquaculture area and has outstanding nitrogen-removing ability (Chen et al., 2023); ultimately, we aimed to determine the transcriptome of this marine microorganism in the presence or absence of nitrogen (potassium nitrate). Then a nitrogen metabolism pathway was constructed based on transcriptome data, and the differential expression of genes encoding nitrogen metabolism–related enzymes was analyzed. The primary objective of this study was to elucidate the nitrogen utilization mechanism of this *K. aerogenes* strain at the molecular level and lay the foundation for studying its adaptation mechanisms in nitrogen-rich seawater.

## 2 Materials and methods

### 2.1 Bacterium and media

*Klebsiella aerogenes* B23 was isolated by Zhanjiang Yuehai Aquatic Seeding Company Limited on Donghai Island, Zhanjiang, China (Chen et al., 2023). The denitrification medium (DM1&2) used in this study was composed of 5.0 g of sucrose, 1.0 g of K_2_HPO_4_, 1.0 g of KH_2_PO_4_, 5.0 g of NaCl, and 2 mL of trace elements (containing MnSO4 1.1 g, MgSO4 1.0 g, CuSO4 1.6 g, FeSO4 1.8 g per liter of distilled water ) per liter of distilled water; these medium was produced either without KNO_3_ (DM1) or with 0.36 g of KNO_3_ (DM2) as the sole nitrogen source. The Luria–Bertani (LB) medium contained 10 g of peptone, 5 g of yeast extract, and 5 g of NaCl per 1 L of distilled water. Additionally, 1 × phosphate buffered saline (PBS) was purchased from BioSharp Biotech (Beijing, China). All culture media were autoclaved at 121°C for 20 min before use.

### 2.2 Experimental design and sample collection

Strain B23 was activated, inoculated into LB broth, and cultured until it reached logarithmic growth phase (OD_600_ = 1.0). After washing with PBS three times, strain B23 samples were resuspended; this bacterial suspension was then inoculated into DM1 or DM2 with 5% (v/v) inoculum, before being sealed with breathable sealing films. The flasks were incubated in a shaking incubator at 30°C for 48 h. Finally, bacterial samples were collected by centrifugation at 24,200 × g for 10 min at 48 h before being stored at −80°C and sent to Majorbio Company (China) for transcriptome sequencing.

After the bacterial suspension was inoculated into DM1 and DM2, growth (OD_600_ value) was measured using an ultraviolet spectrophotometer (UV-3600PLUS, SHIMADZU, China); additionally, nitrogen utilization (total nitrogen, nitrate, ammonium, and nitrite) was tested at 0 and 48 h. The concentrations of total nitrogen, nitrate, ammonium, and nitrite were determined using alkaline potassium persulfate photometry, phenol disulfonic acid photometry, Nessler’s reagent spectrophotometry, and *N*-(1-naphthyl) ethylenediamine photometry (Gilcreas, 1966). All assays were performed in triplicate.

### 2.3 RNA extraction, library construction, and sequencing

Total RNA was extracted from the bacterial culture using TRIzol reagent, following the manufacturer’s instructions (Invitrogen, Carlsbad, CA, USA); genomic DNA was extracted using bacterial DNA extraction kit (Takara Bio, Shiga, Japan). Then, RNA quality was assessed using an Agilent 2,100 Bioanalyzer (USA) and quantified using an ND-2000 spectrophotometer (NanoDrop Technologies, USA). High-quality RNA samples, with an OD260/280 ≥ 1.8, OD260/230 ≥ 1.0, RNA integrity number ≥ 6.5, 23S:16S ≥ 1.0, concentration ≥ 50 ng/μl, and total amount ≥ 1 μg, were selected for subsequent library construction.

RNA libraries for each sample (DM1_1, DM1_2, DM1_3, DM2_1, DM2_2, and DM2_3) were constructed using the TruSeq RNA Sample Preparation Kit (Illumina, San Diego, CA, USA). The Ribo-Zero Magnetic Kit (Epicenter, USA) was used to remove rRNA; then, the mRNA was randomly fragmented into small pieces of approximately 200 bp. Finally, double-stranded cDNA was synthesized using mRNA as the template and random primers (Illumina); this was conducted using a SuperScript double-stranded cDNA synthesis kit (Invitrogen). During synthesis of the second strand of cDNA, dUTP was used instead of dTTP. The resulting double-stranded cDNA was then repaired to obtain blunt ends, phosphorylation at the 5′ end, and an A base addition to the 3′ end. Finally, the cDNA was ligated to a Y-shaped sequencing adapter. The second strand of the cDNA, containing dUTP, was removed using uracil-DNA glycosylase, thereby producing a library containing only the first strand of cDNA.

This cDNA library was enriched and amplified using the Phusion DNA polymerase (New England Biolabs) over 15 PCR cycles. After quantification using TBS380 (Turner Biosystems, USA), RNA-seq paired-end sequencing was performed using Illumina NovaSeq (2× 150 bp). The raw reads generated in this study have been deposited in the NCBI database (accession number: PRJNA984342).

### 2.4 Sequencing data quality control and sequence alignment analysis

Using the Illumina platform, sequencing image signals were converted into text signals via CASAVA base calling and stored in the FASTQ format as raw data. Adapter sequences were removed from reads. Additionally, 5′ ends that contained non-A, G, C, or T bases were trimmed. Reads of low sequencing quality (sequencing quality value < Q20) were also trimmed. Further, reads with an N content ratio of ≥10% were removed. Finally, fragments with lengths <25 bp were discarded after adapter removal and quality trimming, thereby resulting in clean data. Bowtie2 was then used to align the high-quality reads in each sample to the reference genome of *K. aerogenes*.

### 2.5 Function annotation

To obtain comprehensive annotation information for genes and transcripts, genomic sequences were compared using several databases, including Gene Ontology (GO), Kyoto Encyclopedia of Genes and Genomes (KEGG), Clusters of Orthologous Groups of Proteins (COG), Non-Redundant Protein Database (NR), Swiss-Prot, and Pfam.

### 2.6 Differentially expressed gene identification and functional enrichment

The transcripts Per Million reads (TPM) method was used to calculate the expression levels of transcripts in the DM1 and DM2 groups. After obtaining the read counts of these transcripts, differential gene expression analysis between the samples was performed using DEseq2 software (Love et al., 2014). Differentially expressed genes (DEGs) were identified between DM1 and DM2 samples with |log2FC| > 1 and q-value ≤0.05. Then the functions of these DEGs were investigated using cluster analysis, functional annotation (COG, GO, and KEGG), and functional enrichment analysis (GO and KEGG).

### 2.7 Verification of real-time quantitative PCR

qRT-PCR analysis was performed according to the procedure described by Zhou et al. (2022). Primers were constructed using Primer Premier (version 5.0; Supplementary Table S1). The amplified PCR products ranged from 113 bp to 232 bp in length. To estimate the relative expression levels of the DEGs in DM1 and DM2 samples, we used the 2^–ΔΔCt^ method (Livak and Schmittgen, 2001), with actin as a reference marker.

### 2.8 Statistical analysis

SPSS version 21.0 (SPSS Inc., Chicago, IL, USA) was used to perform one-way analysis of variance (ANOVA). Duncan’s new multiple-range test was used to analyze the means, with a significance level of *p* < 0.05. The data presented in the tables and figures represent the mean ± standard error (*N* = 3).

## 3 Results

### 3.1 Nitrogen balance analysis

In this study, nitrogen balance was analyzed by testing and calculating the changes in different nitrogen forms (Table 1). It became clear that *K. aerogenes* B23 growth was significantly hindered when cultured in DM1, which was attributed to the absence of a nitrogen source. Trace ammonium and a slight decrease in intracellular N were observed after 48 h, which may be due to the presence of N in the cytoplasm of some cells. In DM2, the majority of nitrate supplied was utilized by this *K. aerogenes* strain. Specifically, 82.47% of the initial nitrate was transformed into intracellular nitrogen by assimilation, while 16.16% of the initial nitrate was lost through conversion into gas.

**Table 1:** The growth and nitrogen balance of strain B23 during denitrification process (unit:mg/L).

Medium	Time	OD_600_	Nitrate	Ammonium	Nitrite	Intracellular N	N lost by gas
DM1	0 h	0.094 ± 0.007	_	_	_	0.58 ± 0.32	_
	48 h	0.090 ± 0.005	_	0.21 ± 0.08	_	0.37 ± 0.15	_
DM2	0 h	0.087 ± 0.005	50.61 ± 1.13	_	_	_	_
	48 h	1.468 ± 0.006	0.64 ± 0.38	0.05 ± 0.04	_	41.74 ± 1.59	8.18 ± 0.82

–: Undetectable. N lost by gas = Total nitrogen (0 h) – Total nitrogen (48 h).

### 3.2 RNA sequencing and transcriptome annotation

For the DM1_1, DM1_2, DM1_3, DM2_1, DM2_2, and DM2_3 samples, 24.77 Gb of data were analyzed. Overall, we obtained 161,397,874 clean reads with a Q30 score > 94.39% after filtering out low-quality reads and adapters (Supplementary Table S2). Parallelism among the groups was good and sequence alignment was high. All 5,028 genes were determined to match known genes in the listed databases at least once. These Genes were found to have the most hits in the NR (4,994 genes; 99.32%) and Pfam databases (4,477 genes; 89.04%); this was followed by 4,308 (85.68%) in the Swiss-Prot database, 4,258 (84.69%) in the COG database, 3,350 (66.63%) in the KEGG database, and 3,044 (60.54%) in the GO database (Table 2).

**Table 2:** Statistics of annotation results.

Organism	*Klebsiella aerogenes* (enterobacteria)
Genome ID	GCF_007632255.1
Genome Size (bp)	5,249,267
GC Content (%)	54.91
CDS No.	5,028
Genes of NR [Percent(%)]	4,994 (99.32)
Genes of Swiss-Prot [Percent(%)]	4,308 (85.68)
Genes of Pfam [Percent(%)]	4,477 (89.04)
Genes of COG [Percent(%)]	4,258 (84.69)
Genes of GO [Percent(%)]	3,044 (60.54)
Genes of KEGG [Percent(%)]	3,350 (66.63)

The 3,044 genes in the GO database were enriched in biological processes, cellular components, and molecular functions (Supplementary Figure S1). The top three terms in the biological process category were determined to be regulation of DNA-templated transcription (98), transmembrane transport (72), and translation (58). For cellular components, the top 3 terms were integral component of membranes (753), plasma membrane (465), and cytoplasm (417). Finally, for molecular functions, the top three terms were ATP binding (296), DNA binding (271), and metal ion binding (254).

Next, we used the COG database to annotate 4,258 genes, which were then classified into 24 distinct categories (Supplementary Figure S2). Three of these categories, each containing over 400 genes, were identified as follows: (G) carbohydrate transport and metabolism (505, 10.56%), (E) amino acid transport and metabolism (461, 9.64%), and (K) transcription (418, 8.74%). In total, 3,350 *K. aerogenes* genes were assigned to 40 KEGG categories (Supplementary Figure S3). Notably, the most frequently occurring pathways were carbohydrate metabolism (366), membrane transport (327), prokaryotic cellular community (152), translation (83), and antimicrobial drug resistance (70), which were categorized as metabolism, environmental information processing, cellular processes, genetic information processing, and human diseases, respectively.

### 3.3 DEG and gene co-expression cluster analysis

**Figure 1:**
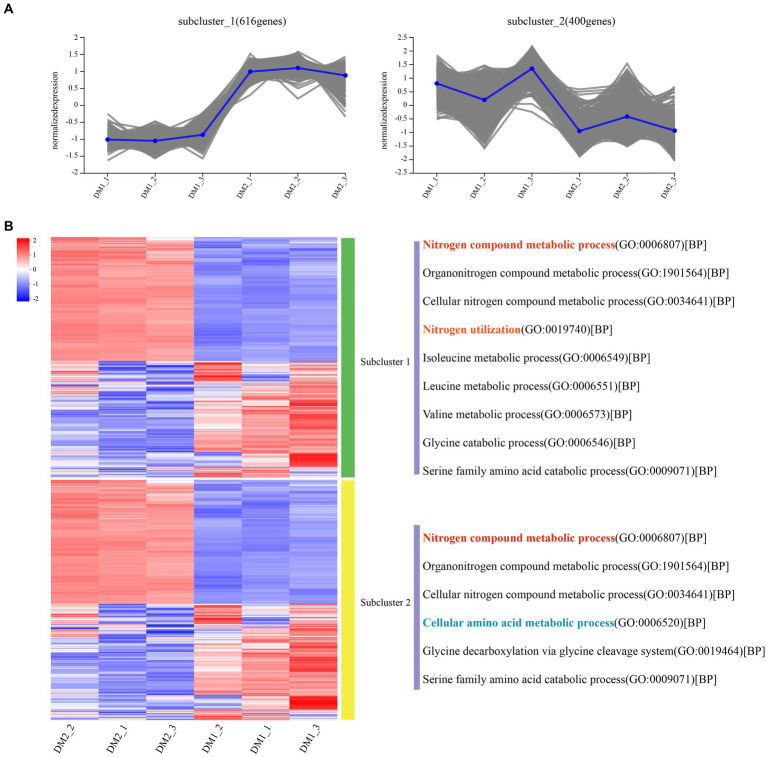
Gene co-expression clusters and heatmap analysis of DEGs in *K. aerogenes*. **(A)** Gene number of co-expression clusters and these gene expression patterns, **(B)** Heatmap and GO terms related to clusters of enriched gene co-expression. DM1_1, DM1_2, and DM1_3 indicate 3 replicates of the control group, while DM2_1, DM2_2, and DM2_3 indicate 3 replicates of the treated group.

Overall, RNA-seq reads from the DM1 and DM2 groups were aligned at an average mapping rate of 96.00% for *K. aerogenes* (Supplementary Table S2). The Pearson’s correlation coefficient between the three biological replicates of the DM1 and DM2 groups showed a strong correlation, ranging from approximately 0.982–1.0 (Supplementary Figure S5). Principal component analysis revealed distinct transcriptomic characteristics between the DM1 and DM2 groups (Supplementary Figure S6); these results suggested that DM2 KNO_3_ treatment significantly affected the transcriptome-wide gene expression of *K. aerogenes*. A total of 1,195 DEGs between the DM1 and DM2 groups were separated into 10 clusters of gene co-expression patterns (Supplementary Figure S4 and Supplementary Dataset S1, S2). Cluster 1 exhibited significantly higher gene expression in the DM2 group than in the DM1 group (control), with 616 upregulated genes in DM2 *K. aerogenes* (Figure 1 and Supplementary Dataset S1). GO analysis revealed that these upregulated genes were associated with the regulation of nitrogen compound metabolism, organonitrogen compound metabolism, cellular nitrogen compound metabolic process, nitrogen utilization, isoleucine metabolism, leucine metabolism, valine metabolism, glycine catabolism, and serine family amino acid catabolism (Figure 1A). Nonetheless, compared to the DM1 group, the 400 genes in cluster 2 showed significantly lower gene expression in the DM2 group (Supplementary Dataset S2). These downregulated genes were determined to be involved in nitrogen compound metabolism, organonitrogen compound metabolism, cellular nitrogen compound metabolism, cellular amino acid metabolism, glycine decarboxylation via glycine cleavage, and serine family amino acid catabolism (Figure 1B).

**Figure 2:**
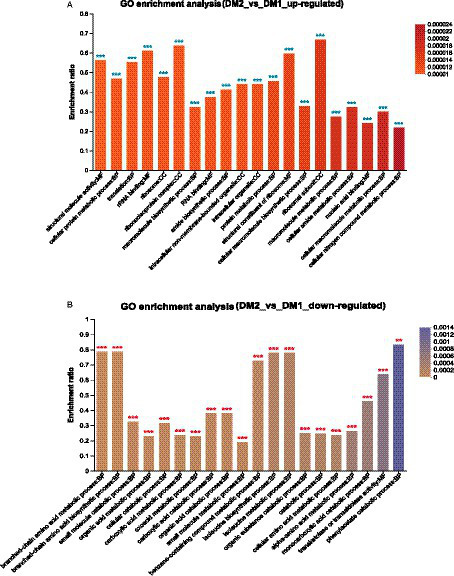
GO enrichment analysis of the DEGs in *K. aerogenes* between the DM1 and DM2 groups. **(A)** Upregulated genes, **(B)** downregulated genes. The label “**” indicates *p* < 0.01, and “***” indicates *p* < 0.001.

Finally, GO enrichment analysis revealed that 4,587 genes were significantly upregulated in the DM2 group compared to the DM1 group. These genes were determined to be associated with ribosomal subunits (GO:0044391), the ribonucleoprotein complex (GO:1990904), rRNA binding (GO:0019843), structural constituent of ribosome (GO:0003735), and structural molecule activity (GO:0005198) (Figure 2A). Alternatively, 1857 genes were determined to be downregulated in the DM2 group compared to The DM1 group; these genes were associated with phenylacetate catabolism (GO:0010124), branched-chain amino acid metabolism (GO:0009081), branched-chain amino acid biosynthesis (GO:0009082), isoleucine biosynthesis (GO:0009097), and isoleucine metabolism (GO:0006549) (Figure 2B).

### 3.4 Nitrate assimilation, arginine metabolism, glutamate metabolism, and ammonia assimilation under nitrogen-rich conditions

**Figure 3:**
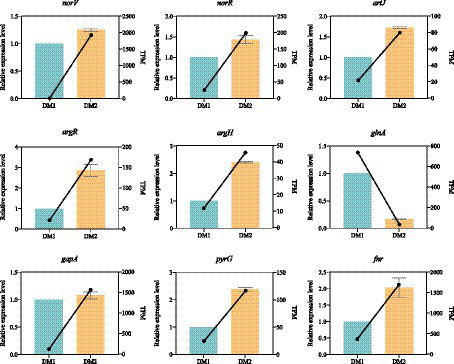
Expression pattern validation of 9 selected DEGs in *K. aerogenes* determined by RNA-seq and qRT-PCR.

Nine candidate DEGs (eight upregulated and one downregulated) were randomly selected; the expression levels of these DEGs were measured using qRT-PCR with specific primers to validate the reliability of the RNA-seq data for *K. aerogenes* under nitrogen-rich conditions (Supplementary Table S1). The expression patterns of the nitrogen treatment–associated DEGs, as observed by qRT-PCR, closely aligned with those from previously established RNA-seq data of *K. aerogenes* (Figure 3); this highlighted the reliability of the RNA-seq data obtained in this study. Additionally, this indicated that the identified DEGs are suitable candidates for further investigation of *K. aerogenes* under nitrogen-rich conditions.

Overall, eight genes were determined to be involved in nitrate assimilation under nitrogen-dependent conditions in *K. aerogenes* (Table 3). Specifically, five of these genes (*norV*/*05380*, *norR*/*05390*, *narI*/*14925*, *FNR*/*RS13560* and *NarK*/*14945*) were upregulated, and three (*narG*/*11865*, *NarK*/*11870* and *ntrB*/*14980*) were downregulated under nitrogen-rich conditions compared to the control. Additionally, in the nitrogen-treated group, five genes involved in arginine metabolism (*artQ*/*16875*, *artJ*/*16870*, *artM*/*16880*, *argR*/*02445* and *argH*/*23940*) were upregulated, while two (*tatE*/*18075* and *argT*/*07020*) were downregulated. Further, under nitrogen-rich conditions, five glutamate metabolism–associated genes (*gshA*/*05525*, *proB*/*19730*, *proA*/*19735*, *gapA*/*09905* and *fdx*/*06275*) were upregulated, while seven (*glnA*/*24240*, *putA*/*15685*, *gltB*/*02550*, *astD*/*10040*, *trpD*/*10200*, *gltS*/*00465* and *ndoA*/*03960*) were downregulated. Finally, in the nitrogen-treated group, five ammonia assimilation–associated genes (*GAT1*/*06645*, *prmB*/*0691*5, *pyrG*/*05015*, *glnS*/*17865* and *Ca1*/*novel0138*) were upregulated, while seven (*glnH*/*17190*, *glnP*/*17195*, *glnQ*/*17200*, *GAT1*/*02860*, *FmdA_AmdA*/*21860*, *arcC*/*14025* and *CA*/*22225*) were downregulated.

**Table 3:** Genes involved in nitrogen metabolism pathways in *K. aerogenes* between the DM1 and DM2 groups.

Involved metabolic pathways	Gene ID	Gene name	Function description	log2 (Fold change)
Nitrate assimilation	FPV33_RS05380	*norV*	Anaerobic nitric oxide reductase flavorubredoxin	9.73
	FPV33_RS05390	*norR*	Nitric oxide reductase transcriptional regulator NorR	2.12
	FPV33_RS14925	*narI*	Respiratory nitrate reductase subunit gamma	2.95
	FPV33_RS13560	*FNR*	Fumarate/nitrate reduction transcriptional regulator Fnr	1.32
	FPV33_RS11865	*narG*	Nitrate reductase subunit alpha	−1.27
	FPV33_RS11870	*NarK*	NarK family nitrate/nitrite MFS transporter	−1.44
	FPV33_RS14945	*NarK*	NarK family nitrate/nitrite MFS transporter	1.50
	FPV33_RS14980	*ntrB*	Nitrate ABC transporter permease	−1.11
Arginine metabolism	FPV33_RS18075	*tatE*	Twin-arginine translocase subunit TatE	−1.52
	FPV33_RS07020	*argT*	Lysine/arginine/ornithine ABC transporter substrate-binding protein ArgT	−1.87
	FPV33_RS16875	*artQ*	Arginine ABC transporter permease ArtQ	1.50
	FPV33_RS16870	*artJ*	Arginine ABC transporter substrate-binding protein	1.01
	FPV33_RS16880	*artM*	Arginine ABC transporter permease ArtM	1.24
	FPV33_RS02445	*argR*	Transcriptional regulator ArgR	2.14
	FPV33_RS23940	*argH*	Argininosuccinate lyase	1.08
Glutamate metabolism	FPV33_RS24240	*glnA*	Glutamate--ammonia ligase	−5.30
	FPV33_RS15685	*putA*	Trifunctional transcriptional regulator/proline dehydrogenase/L-glutamate gamma-semialdehyde dehydrogenase	−1.45
	FPV33_RS02550	*gltB*	Glutamate synthase large subunit	−1.43
	FPV33_RS05525	*gshA*	Glutamate--cysteine ligase	1.74
	FPV33_RS10040	*astD*	Succinylglutamate-semialdehyde dehydrogenase	−1.37
	FPV33_RS19730	*proB*	Glutamate 5-kinase	1.22
	FPV33_RS10200	*trpD*	Bifunctional anthranilate synthase glutamate amidotransferase component TrpG/anthranilate phosphoribosyltransferase TrpD	−1.08
	FPV33_RS19735	*proA*	Glutamate-5-semialdehyde dehydrogenase	1.01
	FPV33_RS00465	*gltS*	Sodium/glutamate symporter	−1.14
	FPV33_RS09905	*gapA*	Glyceraldehyde-3-phosphate dehydrogenase	2.84
	FPV33_RS06275	*fdx*	ISC system 2Fe-2S type ferredoxin	1.75
	FPV33_RS03960	*ndoA*	Non-heme iron oxygenase ferredoxin subunit	−2.69
Ammonia assimilation	FPV33_RS06645	*GAT1*	Type 1 glutamine amidotransferase	2.31
	FPV33_RS17190	*glnH*	Glutamine ABC transporter substrate-binding protein GlnH	−3.20
	FPV33_RS06915	*prmB*	50S ribosomal protein L3 N(5)-glutamine methyltransferase	2.31
	FPV33_RS05015	*pyrG*	CTP synthase (glutamine hydrolyzing)	1.48
	FPV33_RS17865	*glnS*	Glutamine--tRNA ligase	1.48
	FPV33_RS17195	*glnP*	Glutamine ABC transporter permease GlnP	−1.83
	FPV33_RS17200	*glnQ*	Glutamine ABC transporter ATP-binding protein GlnQ	−1.72
	FPV33_RS02860	*GATase1*	Type 1 glutamine amidotransferase	−1.42
	FPV33_RS21860	*FmdA_AmdA*	Acetamidase/formamidase family protein	−1.63
	FPV33_RS14025	*arcC*	Carbamate kinase	−1.36
	novel0138	*Ca1*	Carbonic anhydrase	2.99
	FPV33_RS22225	*CA*	Carbonic anhydrase	−1.29

## 4 Discussion

Microorganism play a vital role in driving the nitrogen cycle, a crucial component of the biogeochemical cycle. Nitrogen fixation is the process by which nitrogen-fixing microorganisms convert atmospheric N_2_ directly into biologically available ammonia; ultimately, this ammonia acts as the primary source of newly supplied nitrogen to the ocean (Fripiat et al., 2021). In the present study, *K. aerogenes* B23 exhibited effective growth in nitrogen-containing medium; additionally, this *K. aerogenes* strain utilized the nitrate provided, with 82.47% of this nitrate being assimilated into intracellular nitrogen. Previous studies have demonstrated that the formation of various enzymes in *K. aerogenes* is dependent on the quality and quantity of the nitrogen source provided in the growth substrate. It is understood that this nitrogen-dependent regulation of enzymes requires the action of the nitrogen regulatory system (Schwacha and Bender, 1993). The *Klebsiella* K312 has been established to convert nitrate to ammonia under nitrate-limited conditions; additionally, this process has been linked to the synthesis of nitrate reductase and nitrite reductase (Dunn et al., 1979). Based on the RNA-seq data of this study, we established how nitrate, from nitrate-containing medium, is utilized and assimilated by *K. aerogenes* B23. Specifically, this *K. aerogenes* strain grown in nitrate-containing medium exhibited unique and distinct expression patterns.

**Figure 4:**
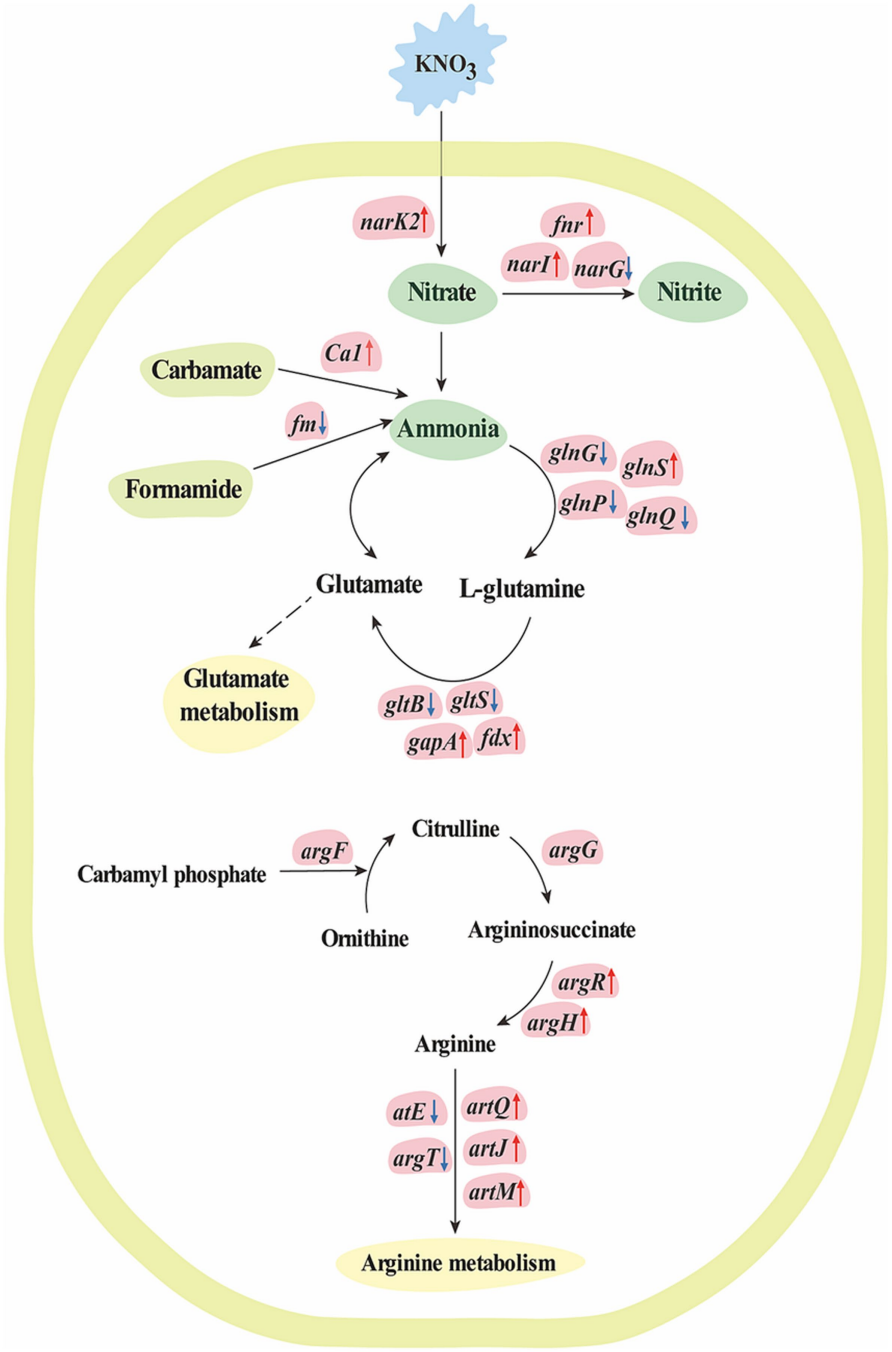
The molecular mechanism for nitrogen metabolism of *K. aerogenes* under nitrogen conditions. The text in blue block indicates *K. aerogenes* was cultured with KNO_3_, while the text in green block indicates nitrogen compound, the text in yellow-green block indicates organic compounds that involve in ammonia metabolism, and the text in yellow blocks indicates metabolism pathways. Up-regulated genes are indicated in pink blocks with up arrow, while down-regulated genes are indicated in pink blocks with down arrow, and non-significant regulated genes are indicated in pink blocks without arrow. Solid arrows indicate direct pathways for transcriptional regulation, while dashed arrows indicate indirect or unclear mechanisms.

In the present study, 1,195 DEGs were found in *K. aerogenes* B23 grown in nitrate-containing medium. Cluster 1 contained 616 highly overexpressed genes in nitrate-treated *K. aerogenes* B23. Then, GO analysis indicated that these overexpressed genes were closely related to nitrogen compound metabolism, organonitrogen compound metabolism, cellular nitrogen compound metabolism, nitrogen utilization, isoleucine metabolism, leucine metabolism, valine metabolism, glycine catabolism, and serine family amino acid catabolism (Figure 1). To better explain the molecular mechanism behind the transport and assimilation of potassium nitrate in B23, we analyzed the associated KEGG metabolic pathways. We constructed the nitrogen metabolism pathway of *K. aerogenes* B23, which included nitrate assimilation, urea cycle (arginine and glutamate metabolism), and ammonia assimilation (Table 3 and Figure 4). Nitrogen metabolism is initiated by the absorption of inorganic nutrients from the environment. Nitrate/nitrite transporter proteins are the components responsible for this transport of nitrate/nitrite into cells for further assimilation (Senior, 1975). Nitrate assimilation is the process by which bacteria convert externally absorbed nitrate into ammonia, which is then used to form organic matter and store energy (Bothe et al., 2007). Nitrate reductase, the initiating enzyme for nitrogen utilization, is widely present in bacterial cells and is a key limiting and regulating enzyme in nitrate assimilation. Specifically, it converts the oxidized forms of nitrogen compounds absorbed by bacteria into their reduced forms, thereby completing the nitrogen metabolism cycle (Jiang and Jiao, 2016). In the *K. aerogenes* B23 transcriptome, we identified the transcriptional isoforms of genes that encode enzymes involved in the nitrate assimilation pathway (Table 3). Additionally, the expression levels of *FNR*, *NarK*/*14945*, and *fdx* were significantly upregulated under nitrogen-rich conditions. During the transition from aerobic to anaerobic growth in *Escherichia coli*, the transcription factor *FNR* plays a crucial role in regulating the reduction of succinate and nitrate (Constantinidou et al., 2006). In denitrifying bacteria, the transport of nitrate and nitrite is typically mediated by transmembrane transport proteins that are part of the major facilitator superfamily, which belongs to the NarK subfamily (Alvarez et al., 2019). In this study, NarK genes were found to be upregulated and involved in nitrate transport within *K. aerogenes* B23. The first step in the nitrate assimilation pathway is catalyzed by nitrate reductase, which converts nitrate to nitrite. However, owing to the toxicity of nitrite to cells, a second step involving nitrite reduction is necessary; this process utilizes ferredoxin and NADH as coenzymes to convert nitrite into ammonia (Ding et al., 2022). In this study, the gene encoding ferredoxin (*fdx*) of *K. aerogenes* B23 exhibited high expression in the presence of nitrate.

Transcripts encoding enzymes involved in ammonia metabolism were found in the *K. aerogenes* B23 transcriptome. Among these, *gshA*, *proB*, *proA*, and *gapA* were significantly upregulated. Glutamine synthetase and glutamate synthase use ammonia to catalyze the production of L-glutamate and L-glutamine, respectively. These amino acids can then participate in glutamate metabolism (Zhou et al., 2020). In the present study, the large subunit of glutamate synthase, *gltB*, was significantly downregulated in nitrogen-treated *K. aerogenes* B23. These enzymes participate in the conversion of ammonia into organic peptides, contribute to the urea cycle, convert ammonia to ornithine to generate arginine, and subsequently catalyze the condensation of the guanidine group on ornithine with the amino group of aspartic acid, thereby forming argininosuccinate (Cunin et al., 1986; Charlier and Bervoets, 2019). Argininosuccinate synthase catalyzes the cleavage of argininosuccinate to produce arginine. Arginase then hydrolyzes arginine to produce urea and regenerate ornithine, thereby completing the urea cycle. Additionally, nitric oxide synthase catalyzes the interconversion of arginine and citrulline to supplement the needs of different amino acids during specific periods of the urea cycle (Mori, 2007; Lorin et al., 2014). In the present study, the gene encoding arginine succinyltransferase, *argH*, was significantly upregulated in nitrogen-treated *K. aerogenes*, resulting in the accelerated breakdown of arginine succinate. Additionally, genes encoding the arginine ABC transporters, *artQ*, *artJ*, and *artM*, were upregulated, which accelerated arginine transportation. The transcriptional regulator *ArgR*, was also upregulated in the nitrogen-treated group; specifically, this protein plays a key role in positive regulation of these arginine ABC transporters. The urea cycle is highly important for bacterial metabolism, with its intermediate products playing important roles in various biological processes. Ornithine and arginine are involved in biosynthesis and metabolism of arginine and citrulline, respectively. Urea in the internal and external environments of bacteria has important physiological significance, especially in facilitating the absorption of external nitrogen sources by plants for growth and development. Further, urea also helps maintain the balance of nitrogen metabolism and recycling within bacterial cells.

In addition to nitrate assimilation and urea decomposition, the glutamate–glutamine cycle can also supply the required ammonia for nitrogen metabolism (Esteves-Ferreira et al., 2018). Specifically, glutamine synthetase converts ammonia into glutamine. Additionally, glutamine synthetase receives the carbon skeleton from alpha-ketoglutarate via glutamate synthase (GLT1/GLTD), thereby allowing the production of glutamate. Finally, glutamate dehydrogenase (GDH1/GDH2) converts glutamate back to ammonia, thereby completing the glutamine synthetase–glutamate synthase cycle. Glutamate synthase is the rate-limiting enzyme in this cycle (Herrero et al., 2001). Nonetheless, *GAT1_2.1* may function as a glutaminase, working in conjunction with glutamate dehydrogenase 2 to break down glutamine and direct 2-oxoglutarate towards the TCA cycle in the presence of excess nitrogen (Kambhampati et al., 2021). In the present study, *GAT1* in *K. aerogenes* B23 was determined to be upregulated under nitrogen-rich conditions and was found to participate in glutamine hydrolysis. Similarly, the upregulation of *prmB* and *pyrG* in nitrate-treated *K. aerogenes* B23 was found to be involved in the hydrolysis of glutamine, whereas *glnS* upregulation was necessary for maintaining glutamine synthesis. Carbonic anhydrase (CA) can also catalyze the generation of ammonia from carbamoyl phosphate, thereby supplementing the consumption of ammonia and generating ATP. In this study, *Ca1* was significantly upregulated in *K. aerogenes* B23 grown in nitrate-supplemented medium. CA catalyzes the decomposition of cyanates into ammonia; moreover, this enzyme can catalyze the reversible hydration reaction of carbon dioxide, ultimately providing CO_2_/HCO_3_^−^ for further enzymatic reactions. Additionally, CA can facilitate energy production by removing CO_2_/HCO_3_^−^.

Denitrification is the process in which microorganisms decompose nitrate or nitrite into N_2_, N_2_O, or NO under anaerobic conditions. It is the main biological process by which reactive nitrogen can return to the atmosphere in gaseous form. NO is a toxic metabolite in bacteria that readily reacts with [Fe-S], ferroheme cofactors, and other transition metal centers (Galloway et al., 2008; Canfield et al., 2010). Bacteria that produce NO typically possess nitric oxide reductase (NOR), which converts NO to N_2_O (Cole, 2018). NOR consists of a small subunit (*NorC*) and large subunit (*NorB*) (Harland et al., 2023). Additionally, *NorV* acts as the primary defense mechanism that aids bacteria in resisting oxidation and nitrite-based sterilization. Further, the *norV* gene has been determined to provide a protective advantage to *Aeromonas hydrophila* against *Tetrahymena* predation, improve bacterial survival within macrophages, and contribute significantly to bacterial virulence in zebrafish (Liu et al., 2019). The NorR regulatory protein detects the presence of NO in *E. coli* and triggers the activation of the genes necessary for NO detoxification under anaerobic and microaerobic conditions (Tucker et al., 2008). In the present study, *norV*, *norR*, and *narI* were determined to be significantly upregulated in nitrate-treated *K. aerogenes* B23; these genes were specifically associated with NO metabolism and, thereby, preventing the toxic effects of NO in this *K. aerogenes* strain.

## 5 Conclusion

Overall, *K. aerogenes* B23 can effectively grow in nitrate-rich culture media and can perform nitrogen fixation, ammonification, nitrification, denitrification, and deamination by upregulating the expression of *FNR*, *NarK*/*14945*, *fdx*, *gshA*, *proB*, *proA*, *gapA*, *argH*, *artQ*, *artJ*, *artM*, *ArgR*, *GAT1*, *prmB*, *pyrG*, *glnS*, and *Ca1*. Additionally, it can mitigate the toxic effects of NO by upregulating *norV*, *norR*, and *narI*. Ultimately, this study reveals a new microorganism that is involved in the marine nitrogen cycle and provides a scientific basis for studying its corresponding nitrogen metabolism mechanisms.

## Data availability statement

The datasets presented in this study can be found in online repositories. The names of the repository/repositories and accession number(s) can be found below: NCBI Database (https://www.ncbi.nlm.nih.gov/), PRJNA984342.

## Author contributions

YC: Formal analysis, Writing – original draft, Methodology. YL: Formal analysis, Writing – original draft. JZhu: Investigation, Writing – review & editing. JZho: Investigation, Writing – review & editing. HL: Conceptualization, Writing – review & editing. YF: Conceptualization, Writing – review & editing. YZ: Writing – review & editing, Supervision.
