# Supplementary material for: Transcriptomic analysis of nitrogen metabolism pathways in Klebsiella aerogenes under nitrogen-rich conditions
Source: Front Microbiol. 2024 Feb 28;15:1323160. doi: 10.3389/fmicb.2024.1323160 (PMC10945327; doi:10.3389/fmicb.2024.1323160)
Supplement: Supplementary file 1 [file Data_Sheet_1.zip › Supplementary Table S1.docx]

| Gene Name | Primer sequences（5'-3') | Sequences length(bp) |
| --- | --- | --- |
| *16SRNA* | F: CTTTATGAGGTCCGCTTGC  R: CGTGTTGTGAAATGTTGGGT | 220  220 |
| *argR* | F: ATAACGATGCGGTGGTGG  R: GGCAGGGGTGGTAAAGATAG | 137  137 |
| *pyrG* | F: CAGGGGCTGGACGATTAT  R: TTTTCAGACCACCGTGTTTC | 196  196 |
| *norV* | F: CGATGAAGTGGACCAGATTG  R: AGGTTGAAGCCGAGGATTT | 117  117 |
| *norR* | F: GCGAACGGGAAAACGATG  R: AACTGGAAATGCTGCGGC | 232  232 |
| *gapA* | F: TCACGCTACCACCGCAAC  R: TCAGGTCAACAACGGATACG | 203  203 |
| *fnr* | F: ACGCATTCAGTCTGGTGGT  R: CCTTGTTCGGTGATGGTGT | 228  228 |
| *artJ* | F: CTGATTGCCGCTCTGCTC  R: CCGACGATTTTGTTGTTGG | 122  122 |
| *argH* | F: GGGCGGGCGTTTTACACA  R: TCAAGGATTTGCTGCGGG | 222  222 |
| *glnA* | F: CCACCAACTCCTACAAGCG  R: TTTCGGCGAGGCAACTAC | 113  113 |
