# Supplementary material for: Transcriptomic analysis of nitrogen metabolism pathways in Klebsiella aerogenes under nitrogen-rich conditions
Source: Front Microbiol. 2024 Feb 28;15:1323160. doi: 10.3389/fmicb.2024.1323160 (PMC10945327; doi:10.3389/fmicb.2024.1323160)
Supplement: Supplementary file 1 [file Data_Sheet_1.zip › Supplementary Table S2.docx]

| Sample Name | Raw reads | Raw Bases (bp) | Clean Reads | Clean Bases (bp) | Clean Error Rate (%) | Clean Q20(%) | Clean Q30(%) | Mapped Reads | Mapped Ratio(%) | Uniq Mapped Reads | Uniq Mapped Reads Ratio(%) |
| --- | --- | --- | --- | --- | --- | --- | --- | --- | --- | --- | --- |
| DM1_1 | 28325856 | 4277204256 | 27915432 | 3328445953 | 0.0239 | 98.45 | 95.26 | 27128566 | 97.18 | 20580602 | 73.72 |
| DM1_2 | 28034648 | 4233231848 | 27389322 | 3155300511 | 0.0241 | 98.35 | 95.05 | 26313194 | 96.07 | 18881956 | 68.94 |
| DM1_3 | 27293896 | 4121378296 | 26978056 | 3262091161 | 0.0244 | 98.26 | 94.68 | 25533418 | 94.65 | 20382026 | 75.55 |
| DM2_1 | 27815630 | 4200160130 | 27203142 | 3139538034 | 0.0245 | 98.18 | 94.69 | 26111406 | 95.99 | 21572342 | 79.3 |
| DM2_2 | 27238326 | 4112987226 | 26847358 | 3455881157 | 0.0245 | 98.19 | 94.68 | 25599018 | 95.35 | 23568115 | 87.79 |
| DM2_3 | 25360178 | 3829386878 | 25064564 | 3129363327 | 0.0248 | 98.09 | 94.39 | 24258491 | 96.78 | 22093888 | 88.15 |
