# Supplementary material for: Transcriptomic analysis of nitrogen metabolism pathways in Klebsiella aerogenes under nitrogen-rich conditions
Source: Front Microbiol. 2024 Feb 28;15:1323160. doi: 10.3389/fmicb.2024.1323160 (PMC10945327; doi:10.3389/fmicb.2024.1323160)
Supplement: Supplementary file 1 [file Data_Sheet_1.zip › Supplementary Table S3.docx]

| Sample Name | Clean Reads | Mapped Reads | Mapped Ratio(%) | Uniq Mapped Reads | Uniq Mapped Reads Ratio(%) |
| --- | --- | --- | --- | --- | --- |
| DM1_1 | 27915432 | 27128566 | 97.18 | 20580602 | 73.72 |
| DM1_2 | 27389322 | 26313194 | 96.07 | 18881956 | 68.94 |
| DM1_3 | 26978056 | 25533418 | 94.65 | 20382026 | 75.55 |
| DM2_1 | 27203142 | 26111406 | 95.99 | 21572342 | 79.3 |
| DM2_2 | 26847358 | 25599018 | 95.35 | 23568115 | 87.79 |
| DM2_3 | 25064564 | 24258491 | 96.78 | 22093888 | 88.15 |
