# Supplementary figures and images for: Transcriptomic analysis of nitrogen metabolism pathways in Klebsiella aerogenes under nitrogen-rich conditions
Source: Front Microbiol. 2024 Feb 28;15:1323160. doi: 10.3389/fmicb.2024.1323160 (PMC10945327; doi:10.3389/fmicb.2024.1323160)

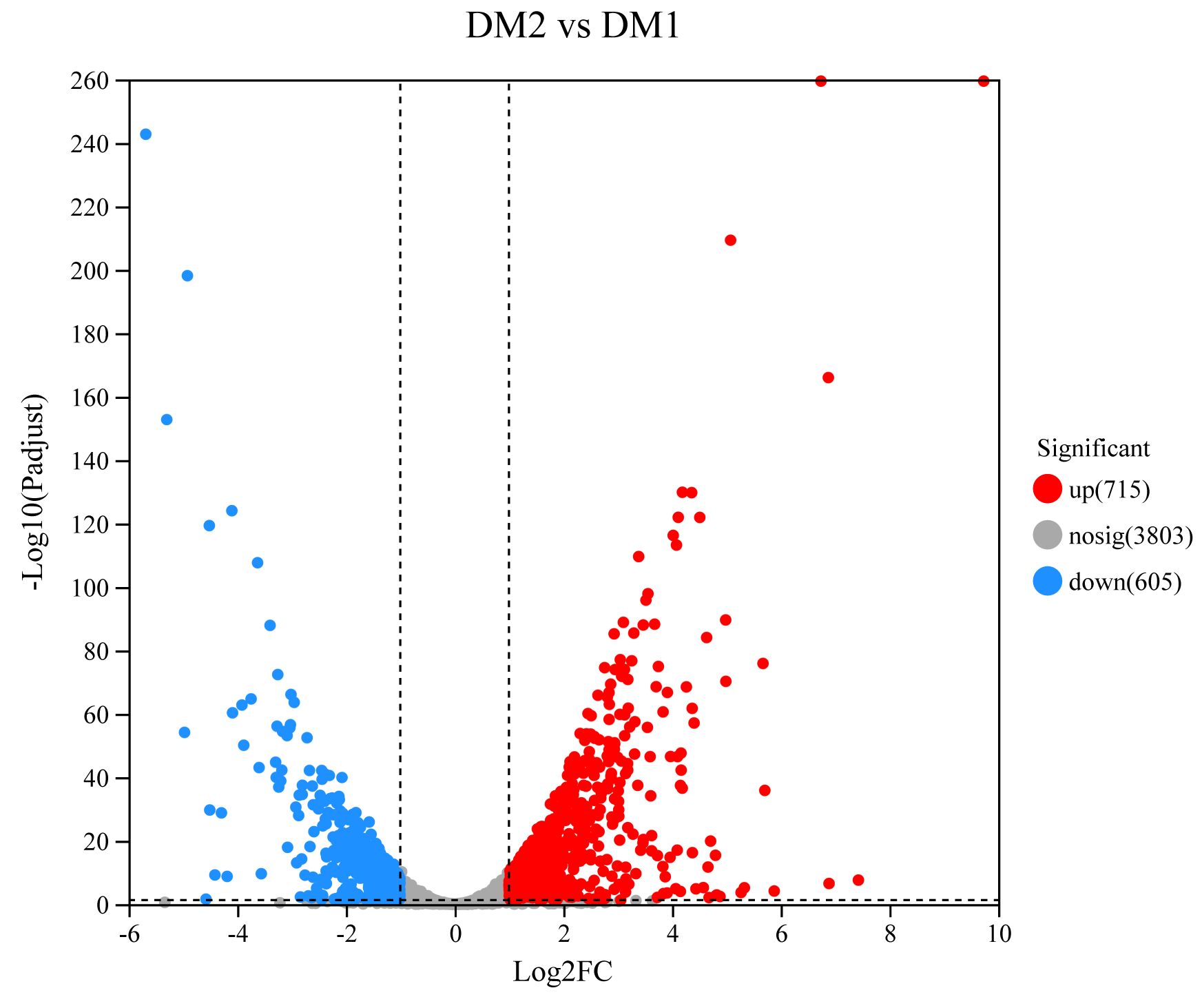

Supplement: Supplementary file 1 [file Data_Sheet_1.zip › Supplementary Figure S4.tif]

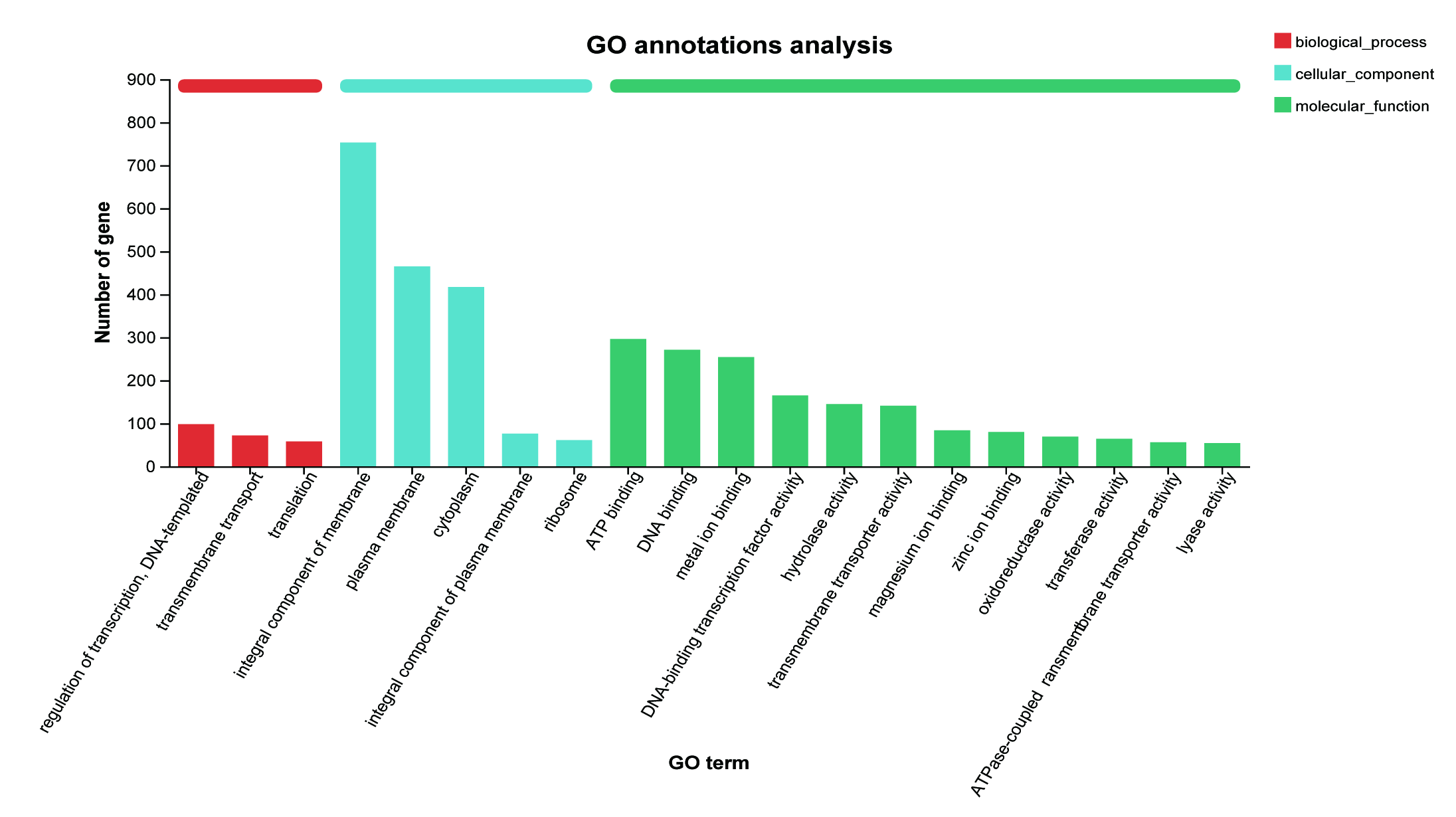

Supplement: Supplementary file 1 [file Data_Sheet_1.zip › Supplementary Figure S1.tif]

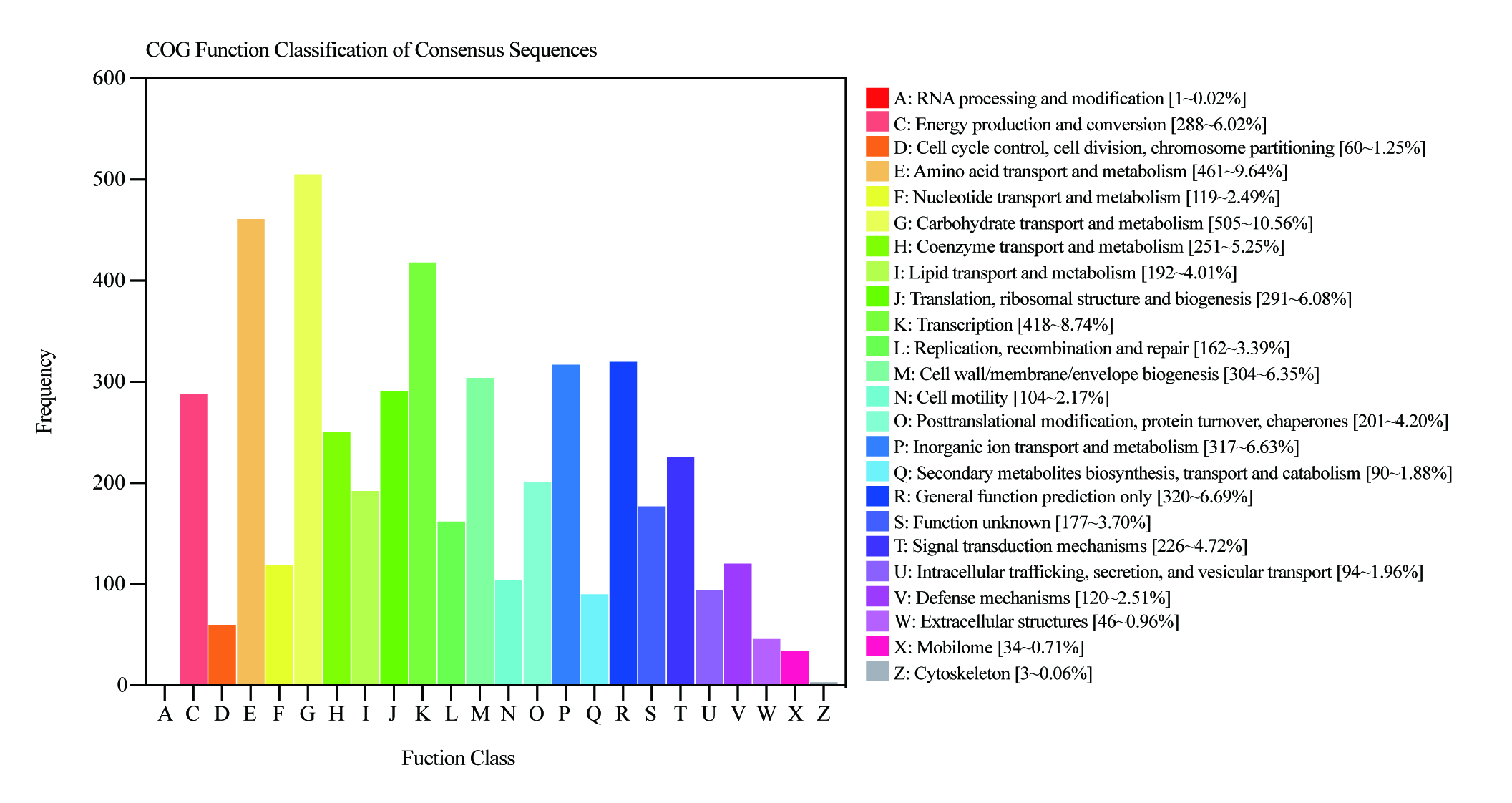

Supplement: Supplementary file 1 [file Data_Sheet_1.zip › Supplementary Figure S2.tif]

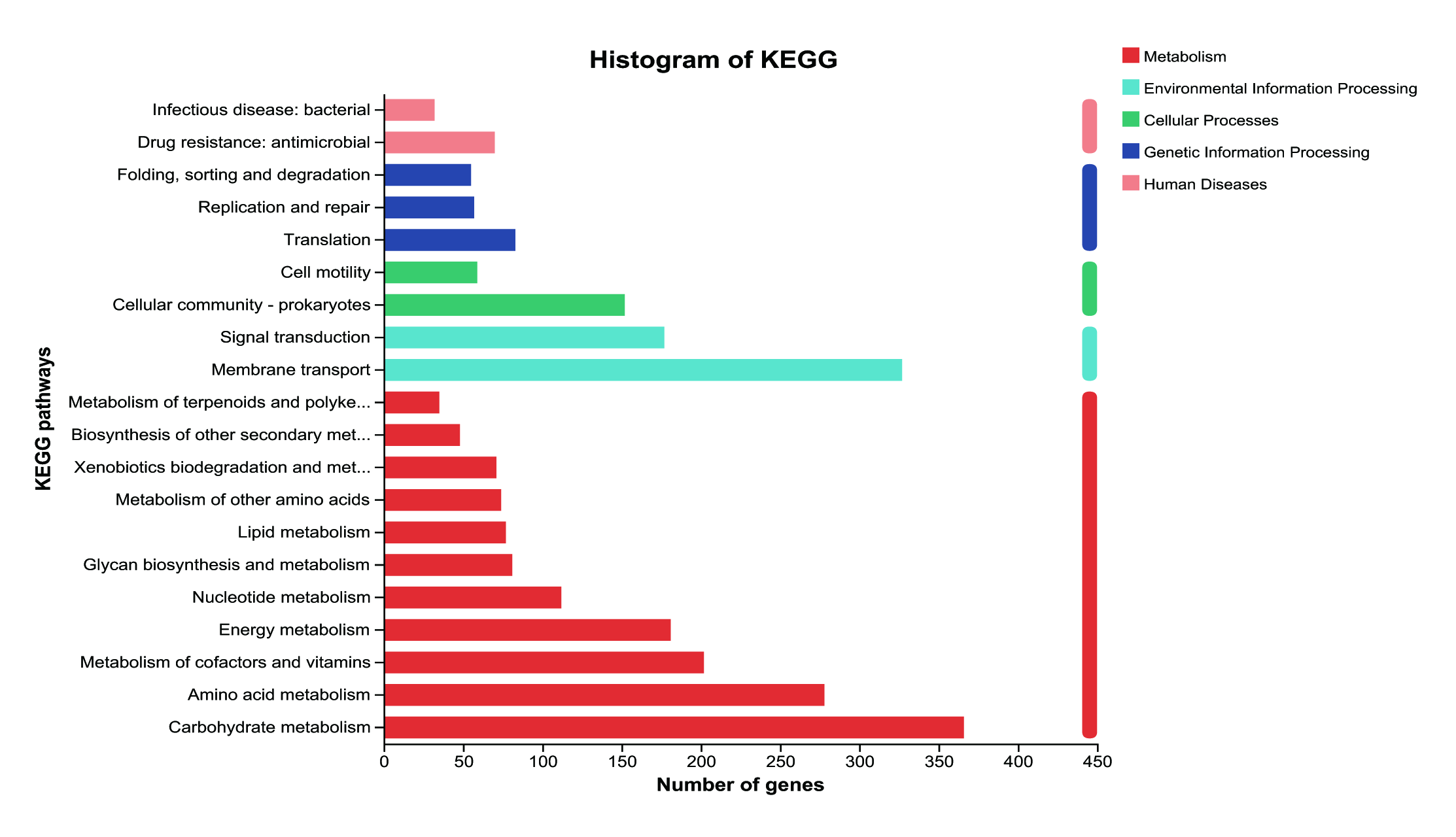

Supplement: Supplementary file 1 [file Data_Sheet_1.zip › Supplementary Figure S3.tif]

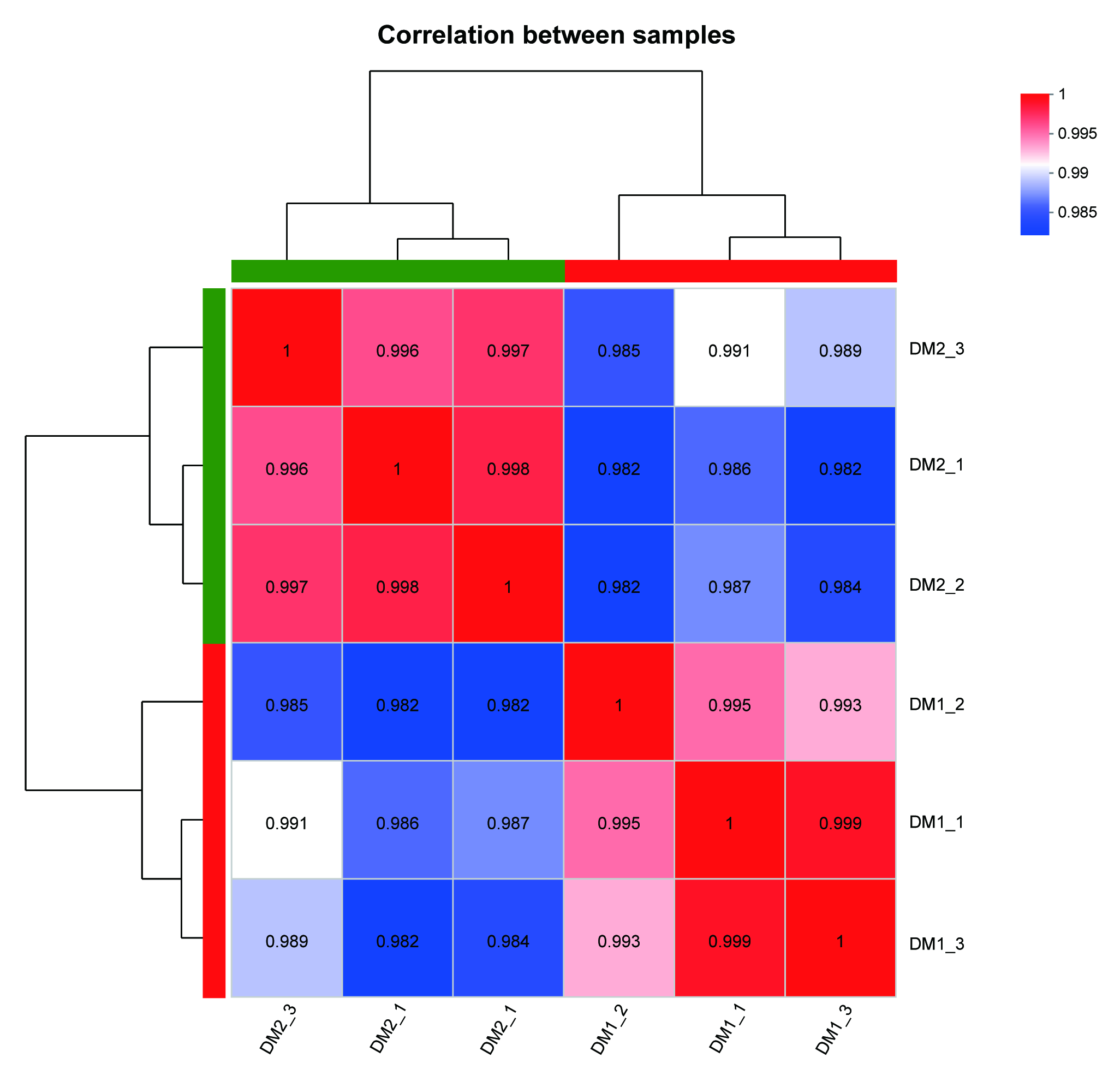

Supplement: Supplementary file 1 [file Data_Sheet_1.zip › Supplementary Figure S5.tif]

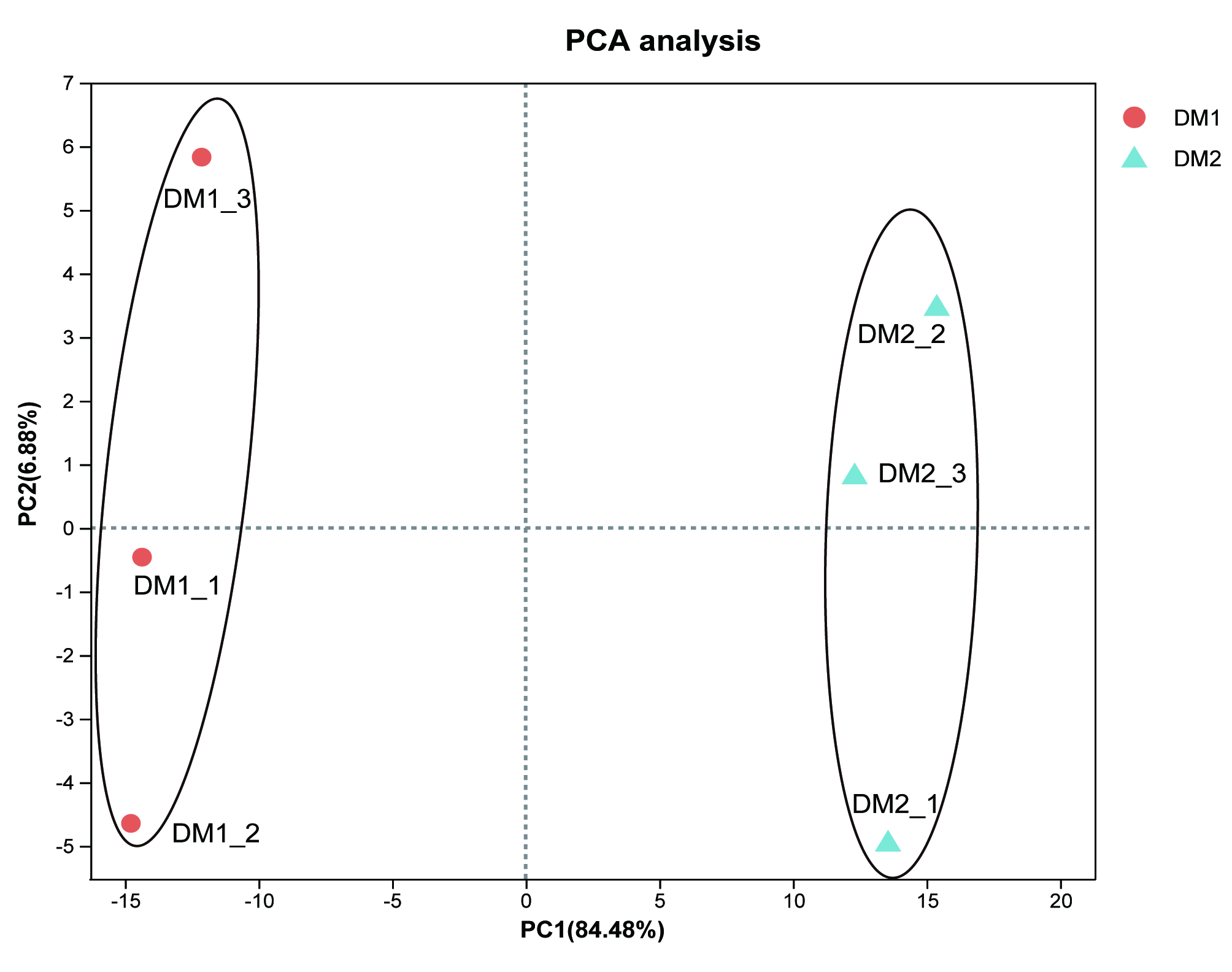

Supplement: Supplementary file 1 [file Data_Sheet_1.zip › Supplementary Figure S6.tif]
